# The Emotional Impact of Family Involvement during Homework in Children with Neurodevelopmental Disorders: A Systematic Review

**DOI:** 10.3390/children11060713

**Published:** 2024-06-10

**Authors:** Amanda Abín, Tania Pasarín-Lavín, Débora Areces, Celestino Rodríguez, José Carlos Núñez

**Affiliations:** Department of Psychology, University of Oviedo, 33003 Oviedo, Spain; abinamanda@uniovi.es (A.A.); pasarintania@uniovi.es (T.P.-L.); arecesdebora@uniovi.es (D.A.); rodriguezcelestino@uniovi.es (C.R.)

**Keywords:** neurodevelopmental disorders, family involvement, homework, emotional variables, contextual variables

## Abstract

Neurodevelopmental disorders can be studied from two distinct perspectives: an internal approach, which examines the causes and consequences of these disorders; and a contextual approach, which considers the role of the family in the lives of children and adolescents. Research has demonstrated that the most significant form of family involvement in families raising a child with NDD is through homework. This involvement has been shown to have an emotional impact on children with neurodevelopmental disorders such as ADHD or dyslexia. The objective of this study is to review published articles on homework and neurodevelopmental disorders, with particular attention to the role of the family and the emotional health of children and families. Method: The review followed the PRISMA guidelines. The final sample consisted of 11 articles, with samples ranging from less than 30 participants to more than 100 at the international level. Results: The results demonstrate the complex methodological and bibliometric picture of the final sample, as well as the many emotional and contextual variables that influence the relationship between homework and neurodevelopmental disorders. Conclusions: Future research should consider how emotional health affects the engagement of families with children with neurodevelopmental disorders.

## 1. Introduction

Neurodevelopmental disorders (NDDs) are a set of conditions that arise from early ages, even before schooling begins. They are defined as difficulties, differences, or deficits in the development of various processes, producing changes at personal, academic, and behavioral levels [1]. NDD may include disorders that affect cognitive and general developmental functioning, as in intellectual disability (ID), or it may include specific disorders such as attention deficit hyperactivity disorder (ADHD), autism spectrum disorder, communication disorders, specific learning disabilities (SLD), and motor disorders [2].

The frequency of NDD varies around the world due to a lack of uniformity in diagnostic criteria between countries, which contributes to variability in incidence and distribution on a global scale. Despite that variability, the estimated prevalence of the most commonly studied neurodevelopmental disorders (NDDs), such as ADHD, has been found to be between 7.9% and 9.5% [3,4], while the prevalence of specific learning disabilities (SLD) is estimated to range from 1.2% to 24% [5,6]. These two are the focus of the present article.

ADHD is a neurodevelopmental disorder characterized by inappropriate levels of inattention, hyperactivity, and/or impulsivity, which presents persistently [4,7,8] and results in social, academic, and occupational difficulties [9]. Furthermore, the presence of comorbidities with emotional or behavioral symptomatology may influence the prognosis [10,11].

SLD represents one of the most prevalent academic needs or difficulties, despite the paucity of research in this area [12]. It is defined as a specific NDD affecting basic school skills such as reading, writing, or mathematics [12,13]. Children with SLD exhibit an average level of intelligence, despite presenting with low reading skills. They face difficulties in comprehending written material, applying grammatical and syntactic rules, organizing information, performing mental calculations, reasoning in mathematical terms, and so forth [14]. As with ADHD, the presence of SLD may co-occur with emotional and/or behavioral difficulties [15].

It is clear that in addition to the academic challenges associated with ADHD or SLD, neurodevelopmental disorders are also linked to the symptoms of anxiety and depression [16,17,18]. A study by Morales-Hidalgo et al. [19] found that NDDs were associated with a higher prevalence and greater severity of concurrent emotional problems than found in people without NDD. Therefore, it is important to identify and treat anxious and depressive symptomatology from an early age, especially in children with NDD. This is required not only to improve mental health but also to reduce academic problems resulting from the emotional situation and to improve family involvement and mental health [19].

Two distinct but complementary approaches have been used to study NDD in children and adolescents. The first focuses on the factors influencing the severity of NDD from an internal or personal perspective, with a particular emphasis on personal and neuropsychological variables [20,21]. The second examines NDD from a contextual perspective [22], investigating the external variables that may influence prognosis, severity, and subsequent development.

The contextual approach allows us to investigate the role of family involvement in the development of children and adolescents with NDD. Given that NDD manifests itself even before children’s schooling begins and that the educational stage is of great importance for their development, family involvement during this period has been extensively researched in recent decades. Various theoretical perspectives and models have been proposed to explain this crucial variable in the academic environment. One of the most prominent models is Overlapping Spheres of Influence [23]. This conceptual framework posits that the child is the center around which all spheres or contexts must interact in order to foster the child’s holistic development. Furthermore, the model emphasizes the significance of collaborative and cooperative relationships between educational institutions, families, and communities. These relationships have been shown to yield a positive emotional benefit for both students and their families [24,25,26,27].

Epstein [28] identified six areas of collaboration, outlining the ways in which families can engage with and be involved in the school and other external social and healthcare actors. Collaboration area 4, also known as “Learning at home”, refers to the support or help that families give their children during homework activities. This area has been the subject of intense debate and controversy in recent years, with homework being defined as the activities that aim to provide students with opportunities to practice what has previously been explained in class so that they can apply those skills and generalize their learning [29].

Family involvement is defined as “the time invested by parents or caregivers in their children’s education” [30] (p. 116). However, when we refer to specific involvement during homework, we can consider it to be parents’ activities at home that are related to the learning that children previously did at school. Furthermore, parental involvement during homework has been demonstrated to have a positive impact on children’s cognitive and language development, as well as their emotional well-being [31]. This involvement has also been linked to improvements in self-esteem, emotional regulation, and perceived competence in academic tasks [32].

This is an acknowledgement that quality family involvement can have a positive influence on students’ emotional development. However, the presence of NDD in children can have a negative impact on their mental health and that of their parents. A study by Cheung et al. [30] found that the presence of an NDD, specifically ADHD, led to a 2.9 times higher risk of parents having mental health difficulties compared to the parents of children without ADHD. Another earlier study similarly found that the anxious and depressive symptoms observed in students with ADHD were comparable to those observed in their parents [33,34].

A number of studies conducted during and following the pandemic have demonstrated adverse effects on parents’ mental health resulting from the presence of neurodevelopmental disorders or specific difficulties in their children. In particular, the mental health of the families of children with NDD has been found to be more adversely affected, with higher levels of stress than in the parents of typically developing children, leading to emotional states such as sadness or depression [35]. This situation has been further exacerbated by the COVID-19 pandemic, as conditions naturally led to high levels of psychological stress in parents [35,36]. Furthermore, the levels of stress and psychological distress appeared to increase in direct proportion to the severity of the behavioral difficulties associated with NDD [35,36]. Therefore, the family’s emotional variables are also an important factor in the study of neurodevelopmental disorders.

### The Current Study

In summary, it is crucial for researchers to analyze neurodevelopmental disorders from a contextual perspective to understand how family involvement during homework can influence a child’s psychological and/or emotional development. This association also takes into account the emotional impact of an NDD on parents. This family involvement at home is essential for any school-aged child but is even more valuable for children with some kind of need or difficulty, such as NDD. It is similarly vital to assess the impact of the emotional health of the family and children facing these challenges in their overall development.

It is currently clear that the study of homework, how it is set, and how it is done by students diagnosed with NDD is a topic with minimal scientific development [37]. Moreover, most studies on the topic have examined samples of the parents of children with normative development, which limits the conclusions that can be drawn from them [38,39,40,41,42,43].

Therefore, the objective of this study is to review published articles on homework and special educational needs. In order to explain the influence of the family during homework in a specific sample, such as children with NDD, we decided to focus on a more general term, “special educational needs” (SEN) [44,45]. This term was included in order to study children with neurodevelopmental disorders, which indicates that they present a significant severity level that affects their family and school environment, as well as having emotional effects.

The articles selected for analysis were chosen based on two main criteria: the age of the sample, which had to be in elementary or secondary education, and the publication date, which had to be within the last 10 years (between 2012 and 2023). Furthermore, the study aims to contribute to the understanding of SEN, with a particular focus on neurodevelopmental disorders and the internal and contextual variables that may influence this relationship. The study also aims to shed light on the emotional symptomatology that may be present in these disorders and the role of the family during homework. This will enable the identification of specific implications at the academic, personal, health, and emotional levels, which will help improve parental involvement during their children’s homework, particularly in the context of NDDs such as ADHD and SLD.

## 2. Method

The study used a retrospective observational research design. The literature review was based on the criteria in the PRISMA statement (an updated guideline for reporting systematic reviews) [46] in order to systematically identify and examine relevant studies. The objectives of this review were approved by the ethics committee of the Principality of Asturias with the reference code “Ethical Research 70/19” following the PRISMA guidelines.

The systematic review included studies that (a) analyzed the relationship between homework and SEN. It should be noted that the term SEN was included as an inclusion criterion for children with NDD that influence the different areas of their lives; (b) had a sample of students in elementary and/or secondary education; (c) were published in Spanish or English in databases such as SCOPUS and WOS; (d) and finally, were published in the last 10 years, between 2012 and 2023.

**Figure 1:**
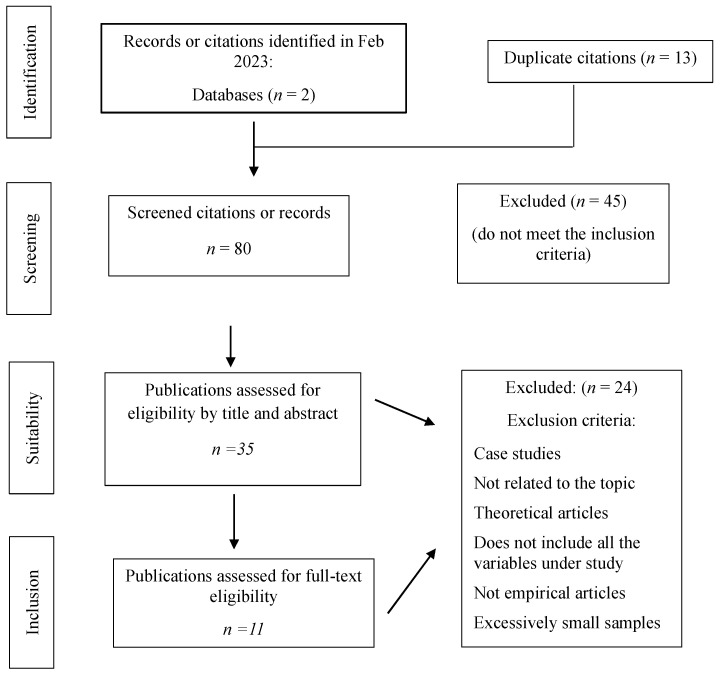
PRISMA flowchart of the systematic review. Adapted from Page et al. [46].

Figure 1 summarizes the selection process for the sample of articles, as well as the results, including the justifications for exclusions during the full-text screening phase. The bibliographic search was conducted during February 2023, specifically between 2 and 4 February 2023, in the main psychology and education databases such as the following: SCOPUS and Web of Science. In WOS, the search was also refined by Indexed Journals, selecting Science Citation Index Expanded (SCI-Expanded) and Social Sciences Citation Index (SSCI). Boolean search terms were used in the title, abstract, and/or keywords: (learning disability* OR learning difficult* OR SEN OR special educational needs) AND (homework). In all the databases, the search was restricted to the period 2012–2022.

In the first stage, all possible results were considered, and duplicates between databases and searches were eliminated. In the second stage, the titles and abstracts were read to refine the list, excluding those that did not refer to the subject of the review or did not meet the previously established criteria. Finally, the complete texts were reviewed to produce the final sample.

The search results were entered into the EndNote bibliographic manager to eliminate duplicates and work with the information about each article.

### 2.1. Inclusion and Exclusion Criteria

The criteria to include the articles were that (a) the articles were published between 2012 and 2022; (b) the study samples were elementary- or secondary-school age; (c) they examined the relationship between homework and pupils’ educational needs; and (d) they were published in English or Spanish.

Studies were excluded according to the following criteria: (a) studies published as a book chapter or conference abstract and not as a scientific article; (b) case studies or studies with excessively small samples; and (c) meta-analyses and reviews.

### 2.2. Data Collection

Following the PRISMA criteria [46], the two databases noted above were used in the identification phase, yielding a total of 93 articles from the search expression and publication filters (years 2012 to 2022). Each extracted article was then reviewed against the inclusion and exclusion criteria established above. This phase produced 13 duplicates which were eliminated before the screening phase, leaving a total of 80 pre-selected articles.

In the screening phase, 45 of the 80 pre-selected articles did not meet the proposed inclusion criteria, either because they did not deal with the variables mentioned, they were not scientific articles, or because they had excessively small samples. This meant a total of 35 articles were selected.

The review of suitability against the exclusion criteria showed that 24 articles did not meet our inclusion criteria for various reasons: they were case studies, not specifically related to the topic, theoretical articles, or meta-analyses, did not include all the variables under study or the variable relationships were not clear, or the samples were excessively small.

Finally, after the inclusion phase, only 11 articles that met all the criteria were included, which we analyze below.

## 3. Results

The following categories were recorded as most relevant to the analysis and extraction of results: (a) year of publication, language and publication journal; (b) variables related to the sample analyzed (age of the sample, sample size, sample country, special educational needs of the sample); (c) informant, and data collection method; (d) emotional variables, homework and students with NDD; (e) impact of individual and contextual characteristics on homework performance in children with NDD; (f) interventions to enhance the abilities associated with NDD and their execution during homework.

### 3.1. Year of Publication, Language and Journal

Since 2012, research on the topic has been scarce. From 2015 to 2020, only one article was published per year, with three publications in 2012 and two publications each in 2021 and 2022. The 11 selected articles were published in English in nine different scientific journals. Only the Journal of Attention Disorders and Learning Disabilities Research and Practice published more than one article in the last 10 years. The rest published a single article each during the decade (Table 1).

### 3.2. Variables Related to the Sample Analyzed

The samples used in the studies varied widely. We divided them into 4 groups: between 1 and 30, between 31 and 100, more than 100, and more than 1000. The most common sample size range was the second group (36.36%), between 31 and 100. This was followed by the third group (27.27%) of samples larger than 100 subjects, while groups 1 and 4 were the same percentages (18.18%).

Looking at each sample’s special educational needs, 18.18% had ADHD with some type of comorbidity; 27.27% had SEN but not defined; and 54.5% had either learning disabilities or ADHD. Therefore, 72.68% had neurodevelopmental disorders.

In addition, 81.82% of the sample attended primary education (6–12 years old) compared to 18.18% who were in secondary education (12–16 years old).

Most of the samples were from the USA (54.55%), with other countries only appearing in a single article each (China, Cyprus, Germany, Israel, and Italy) (Table 1).

### 3.3. Informant and Data Collection Method

The selected articles used different information and data collection methods. However, three articles (27.27%) addressed the three spheres involved in academic life (student–family–teacher).

Just under half of the data collection (five articles; 45.45%) was done via questionnaires or scales, although questionnaires were also combined with psycho-pedagogical tests in 27.27% of the cases. Intervention tools were used in two articles (18.18%) and only one article used school records linked to questionnaires and psycho-pedagogical tests (9.09%) (Table 1).

### 3.4. Main Findings of the Studies Analyzed

The main results of the studies selected for this review are presented in the following table (Table 2)—previously used by Álvarez-García et al. [47]—together with the rest of the information classified as relevant to the study. This template is used to summarize each article’s title and authors, the year of publication, the journal it was published in, the objective of the study, the variables studied, sample and age of the sample, the country of the sample, and main results related to the objective.

### 3.5. Emotional Variables, Homework and Students with NDD

Emotional variables, such as stress, anxiety, and depressive symptoms, were addressed in 45.45% of the selected articles. In addition to focusing on aspects related to mental health, the articles attempted to indicate how homework can influence certain emotional states. This is even more pronounced in the students with NDD since their difficulties and needs mean that they may suffer greater personal, social, and educational maladjustment or even experience negative emotions such as frustration and stress at not being able to achieve the goals set for them in the school environment.

In this regard, Xiao et al. [57] found that students with dyslexia had higher scores on anxious and depressive symptomatology, assessed with the Screen for Child Anxiety Related Emotional Disorders (SCARED) [59] and the Children’s Depression Inventory-Short Form (CDI-S) [60]. They also wanted to determine what kind of relationship existed between dyslexia and anxious/depressive symptoms, considering the role of homework. In the case of dyslexia–anxiety, they found no direct relationship. However, they did find a direct relationship between dyslexia–depression. Conversely, stress acted as a mediating variable, so dyslexia could cause stress-related symptoms, which could in turn cause depressive and/or anxious symptoms. In the case of homework, a variable of great interest in our review, they found dyslexia caused students to take longer to do homework, which could lead to anxious and/or depressive symptoms.

As previously stated, children’s and families’ emotional health is of paramount importance for a child’s optimal overall development [35,36]. This is also significant when children who have neurodevelopmental disorders do homework. This was of interest to Touloupis [55], who decided to investigate family involvement in homework during the COVID-19 pandemic. Following a study with 271 families and their children, the conclusion was that the families of children with SLD tended to show less beneficial ways of helping during homework and this type of involvement was even more visible and frequent when parents exhibited greater fear of COVID-19 and a low sense of resilience. In line with other authors, the study concluded that the families of children with SLD who scored high on the “fear of COVID-19 through the fear of COVID-19 Scale (FCV-19S)” [61] and a “low sense of resilience” measured by the Connor–Davidson Resilience Scale (CD-RISC) [62] were more likely to show less beneficial family involvement during homework, which could produce a more conflictual family environment, less use of patience or beneficial methods, emotional problems arising from fear and stress caused by the situation, etc., [63,64,65,66]. In turn, the parents of children with SLD also showed higher levels of stress and more feelings of shame, guilt, and frustration of needs during family involvement in homework compared to the parents of students without SLD [35,36,49,58]. This seems to increase the likelihood of adopting a less beneficial form of family involvement during homework, with parents controlling and exerting pressure on their children, interfering, and giving orders during the time they participate in homework [53]. In fact, taking a study conducted by Booster et al. [48] on ADHD, homework, and comorbid disorders as a reference, students with ADHD and an internalizing and/or externalizing disorder presented more problems with respect to the execution and completion of homework tasks. This was assessed by the Diagnostic Interview for Children and Adolescents-Revised (DICA-R) [67], ADHD Rating Scale-IV (ADHD-RS-IV) [68], the Behavior Assessment System for children (BASC-2) [69], the Wechsler Individual Achievement Test (WIAT) [70], and The Home Observation for Measurement of the Environment (HPC) [71]. This is extremely important since, in addition to the presence of the attentional disorder, comorbidity with other emotional and motivational disorders can impair homework completion, leading to the use of behavioral strategies that are not adaptive during this process, and damaging children’s relationships with their parents [48].

### 3.6. Impact of Individual and Contextual Characteristics on Homework Performance in Children with NDD

It has been demonstrated in research that the homework performance of students with NDD may vary depending on certain individual or contextual characteristics. This was addressed in two of the selected articles, corresponding to 18.18% of the sample.

In terms of *individual characteristics*, studies suggested that differences in homework performance may be found depending on ADHD diagnostic status and comorbidity status (with dyslexia or internalizing/externalizing disorders).

The objective of a study by Mautone et al. [50] was to understand how homework performance might change depending on these individual characteristics. They used the *Homework Performance Questionnaire* (HPQ) [72], a questionnaire completed by parents and teachers, composed of three family factors—student task orientation/efficiency, student competence, and teacher support—and two teacher factors—student responsibility and student competence.

Specifically, Mautone et al. [50] found that the scores on the HPQ-P were specific and significantly related to the measures of homework behavior, academic skills, and parent–teacher relationship quality. Moreover, parent-scored student task orientation/efficiency was significantly and positively related to teacher-scored student responsibility. Parent-scored student competence was positively related to teacher-rated student competence and academic achievement. As for the teacher variables, teacher support rated by parents was significantly related to parents’ perceptions of the quality of the parent–teacher relationship.

The sample of students with ADHD—either with comorbidity or with a monodiagnosis—were expected to have lower scores rated by teachers and families than students without. The study reported significant differences between children with and without difficulties, and that in addition, students with ADHD-dyslexia comorbidity received lower student competence scores than children with an ADHD monodiagnosis.

It is true that the focus of this research was on a purely academic level, with a specific sample. Nevertheless, it is important to highlight that in a comparable cohort of children with ADHD, homework performance may be negatively impacted by anxiety disorders. Indeed, the most prevalent comorbidity of ADHD is with internalizing disorders [17,73,74]. The coexistence of an anxious disorder may result in diminished performance in educational tasks, such as homework [75].

Another influential characteristic is *contextual since* it is related to family involvement in homework. This involvement is what causes students to complete their homework and also significantly improves their performance because it encourages better study habits, generates positive feelings towards academic tasks, improves their emotional development, and supports their self-esteem [31,32,56,76]

In some studies [54,74], ordinal logistic regression indicated that certain individual and contextual variables influenced the quality and type of involvement. One of these individual variables was the POC or “parents of color” variable. The authors found that non-white parents were less likely to be involved in homework. In fact, it seems that this involvement existed but only in the family environment, without extending to the school.

Another variable referred to effective collaboration between school, family, and community, as explained in Epstein’s model [23,28]. This improvement-oriented collaboration with children with NDD has been demonstrated to lead to positive outcomes in their holistic development [24,77].

### 3.7. Interventions to Enhance the Abilities Associated with NDD and Their Execution during Homework

Due to the great debate surrounding homework, various interventions and methods to improve how it is implemented have been performed, considering some of the personal and contextual variables mentioned above. Just over a third (36.36%) of the selected articles were along these lines. Of the four intervention programs identified in the results, only two addressed emotional aspects, the study by King-Sears et al. [53] and the study designed by Multhauf et al. [52].

One of these interventions was a program called *Child Life and Attention Skills* (CLAS) [78], which consisted of three empirically supported behavioral interventions tailored to children with ADHD inattentive subtype (ADHD-I) and with comorbidity between ADHD and SLD. The objective was to carry out behavioral training with the parents, as well as training the child’s academic skills and relying on daily reports from the teacher. The authors looked at a sample of 74 children with an ADHD-I monodiagnosis and the intervention with CLAS, showing that it is an effective program for improving performance and organization during homework in students with neurodevelopmental disorders, although there were differences between the students with ADHD-I and the students with comorbid ADHD and SLD. For example, although all the children showed large improvements after the intervention, the children with comorbid SLD and ADHD-I demonstrated greater problems with homework than those with an ADHD-I monodiagnosis. The intervention led to the conclusion that the optimal emotional state for both parents and children with NDD is one in which positive emotions are present, stress and other anxious symptoms are reduced, and there is a high level of intrinsic motivation.

Another of the interventions focused on new technologies, in this case, applied to chemistry in secondary school. The study used a methodology focusing on the use of Pencasts using the Livescribe Smartpen. A Pencast is an interactive document that can be accessed online and allows students to listen and view handwritten notes and recorded audio prepared by the teacher to work on content. Smartpens are equipped with a microphone for audio recording and an infrared camera that simultaneously tracks the written content or drawing on microdot paper, matching it with the verbal content. The students would therefore have access to all this visual and verbal content to review what they need and do their homework more effectively using this technology. Pencasts for homework problems allowed students with and without NDD to flexibly use the same technology to individualize practice and review content outside of school hours. The methodology was applied to 19 students, 4 of whom had NDD, and the results show that the students benefited from using Pencasts to enhance learning and improve performance in chemistry. The students with NDD specifically used Pencasts regularly, which helped them work on organization, self-regulation, access to content, and review skills. Moreover, the teacher’s records showed that the homework completion rate was 80.5%, also improving the score on that homework, leading to the conclusion that using electronic techniques to do homework is an effective methodology for improving academic results and motivation [53].

Considering the importance of homework design and the accompanying feedback as a necessary condition to foster interest and autonomous motivation during homework completion, an intervention called Radical Raceway (RR) was developed that focuses on gamification components. It focuses on small group work and uses mystery motivators and interdependent group contingencies.

The study implementing the Radical Raceway intervention used a sample of 24 students with IEPs and sought to examine the effects of this multicomponent intervention to see how it might influence homework completion and accuracy. The results confirmed its effectiveness, as homework completion and accuracy increased immediately and markedly. Furthermore, various studies have shown that RR is an effective way of improving homework performance for students both with and without special needs [51,79]. Moreover, for the students with some type of need and who have IEPs, a significant improvement was also seen when using RR. Finally, there was social acceptance from teachers as they felt that it was an intervention that required little time or effort and significantly improved students’ performance on their assignments. Similarly, the students also rated RR as an important strategy and a procedure they liked [51].

As already mentioned, the family is a cornerstone of children’s education. The need to provide families with information about different educational needs in order to improve their involvement during homework is now well known. Specific training for the parents of children with dyslexia was addressed in the article by Multhauf et al. [52]. This group-based cognitive–behavioral intervention for the parents of children with dyslexia proved effective as it improved parental stress, dyslexia-related specific stress, and parental competencies to be beneficially involved in homework. The study involved a sample of 39 mothers of only children with dyslexia with an experimental group that received the intervention on emotional and motivational aspects and a control group that did not.

The results regarding parental competencies in dealing with dyslexia are particularly encouraging because the data indicate the effectiveness of the program, which was observed by assessing the mothers with the Parenting Stress Index (PSI) [80] and an ad hoc questionnaire designed to measure the specific effects of the training. The results provide important information. For example, the effects of the intervention were consistent regardless of where the homework was done, although the mothers showed higher levels of stress if their children only did homework at home, as they needed to spend more time and effort on the completion of these tasks with appropriate strategies. Where this intervention was implemented, mothers felt more confident in facilitating literacy acquisition and acquired techniques to motivate their children during homework, in addition to reducing their stress levels during homework with improved parental competencies [52,81].

## 4. Discussion

This paper reviewed the studies published from 2012 to February 2023 on homework and pupils with neurodevelopmental disorders in both elementary and secondary education in order to determine the variables influencing this relationship, the individual and contextual factors that could mediate it, and the relationship between the two concepts, also considering methodological characteristics and bibliometric properties. The results show that scientific production on this subject has been rather scarce, and in fact, one might say that scientific research on the relationship between homework and neurodevelopmental disorders is non-existent in Spain and is deficient at the European level, where only four articles have been published in the last 10 years. This is an important gap, as more studies with samples from more countries are needed, as well as more research on the subject at the European level, where homework has been the subject of great debate and social and educational interest. In addition, it is important to highlight that there is far less research if we restrict ourselves to variables referring to SEN or NDD.

After analyzing the 11 articles identified for our study, it is worth mentioning the prevalence of neurodevelopmental disorders such as ADHD and SLD, sometimes occurring in comorbidity both with each other and with other internalizing and externalizing disorders. This conclusion, in relation to homework, has allowed different studies to affirm that many students consider homework to be a “chore” [82] and it can even be an issue that causes conflict at home [29]. In relation to this, students with NDD were found to have higher levels of stress related to homework compared to students without needs [53].

In this regard, self-determination theory [83] posits that the good emotional state of both parents and children produces positive emotions and autonomous motivation during task performance. This becomes even more important when there is an NDD. As previously reported, the presence of NDD in children is associated with an increased risk of parental anxious and/or depressive symptomatology [33,34]. In this regard, recent research has demonstrated that the more stressed the parents of these students were, the more they perceived that their needs were frustrated while helping with homework, and therefore, the more they felt ashamed, guilty, and stressed [84].

What we saw from the selected studies is that, on the whole, this parental involvement in homework, when it is good support and guidance, free from harmful emotions, becomes even more important for students with NDD, and is a necessary variable for children’s school performance, as it results in a positive emotional state and an increase in intrinsic motivation [49,85]. Furthermore, in relation to the support that children with NDD require, it has been claimed that children with SLD tend to require more help from their parents compared to peers without SLD [86].

It is also important to increase the amount of research in secondary education, as most of the articles we identified used elementary-school samples (81.82%). Although assigning homework in secondary education has different characteristics from elementary-education homework, it is still part of compulsory schooling and therefore more research with children aged 12 to 16 would promote an understanding of the influential variables at this stage of schooling and would also allow us to determine differences in contextual variables such as family involvement.

Only two articles from our sample reported using a secondary-school sample and both referred to methods or intervention strategies that improve homework performance. Authors such as Mendicino et al. [87] have stated that digital systems that provide tutoring, modeling, scaffolding, and feedback about processes and errors positively influence learning and homework completion. Despite this, interventions that aim to improve homework execution in students with NDD do not tend to focus on emotional aspects such as those discussed in the present article.

One of these programs is Child Life and Attention Skills (CLAS) [78]. The study assessing it demonstrated significant improvements in academic aspects such as attention, social skills, organization, and overall functioning rated by teacher; contextual aspects such as organization during homework rated by the family; and especially in emotional aspects such as the realization that positive emotions, reduced stress, and good motivation are needed for the rest of the consequences to be possible [78]. It is used with students with ADHD inattentive subtype (ADHD-I) and with comorbidity between ADHD and SLD and tries to break away from traditional ADHD interventions that only focus on problem behaviors, seeking to reduce hyperactivity and impulsivity. These results indicate the need for intervention strategies and programs to work on comorbidities in NDD, such as ADHD and SLD.

The other program concerning work on emotions is aimed at working from the contextual point of view, given the potential influence of NDD on parents’ mental health and the impact of this parental mental health on the development of children with NDD. The aforementioned intervention [52] enabled the mothers of children with SLD, such as dyslexia, to reduce their maternal stress related to both NDD and homework completion, and to increase their competencies for coping with their children’s personal, academic, and emotional challenges.

The two programs most closely related to the academic impact of homework, Radical Raceway [51] and Pencasts [53], produced the following insight. It can be demonstrated that a supplementary intervention, which is specific to NDD, is necessary to address the multiple underlying deficits, including those related to behavioral and emotional domains [54].

In conclusion, it is crucial to recognize that homework is not solely a component of the academic environment; it is primarily conducted within the home environment, where parental involvement is of paramount importance. In the context of children with neurodevelopmental disorders, it is particularly important to prioritize emotional aspects, given their influence on both children and parents, and the potential for anxiety and depressive symptoms to manifest in a direct manner.

## 5. Conclusions

The results of this review have significant implications for the field of research on neurodevelopmental disorders. This becomes even more pertinent when we consider the paucity of research in this area, particularly in relation to the widely debated and important topic of homework and the role that parents play in this regard, from an emotional and psychological perspective. For this reason, the practical implications at educational, family, policy, and mental health levels are discussed.

The results of the present review indicate that emotional state is a priority in the educational sphere. Pupils with neurodevelopmental disorders are often found to exhibit compromised emotional states, which are frequently associated with comorbid disorders such as anxiety and depression. In this regard, educational professionals need to be trained in mental health in order to be able to prevent this symptomatology at a general level, but especially for pupils with some educational needs. It is in the educational environment that quality involvement with the family must be sought, focusing on quality two-way communication that allows the generalization of such interventions at an emotional level to cope with academic demands at home, for example through homework. When it comes to homework, it is clear that setting homework is universal in many countries, irrespective of the presence of a neurodevelopmental disorder. Homework not being adjusted to the needs of the pupil can give rise to emotional difficulties, which in turn can result in increased stress and anxiety, negatively impacting performance. Consequently, education professionals must receive training to enable them to tailor homework to individual characteristics and needs.

It is important to bear in mind that NDDs in children can also cause a certain emotional maladjustment in parents, which in turn leads to a parenting style that is not beneficial for the child’s development. Consequently, it becomes even more important to develop intervention programs aimed at families so that they can adjust their emotions to the needs of their children and acquire the necessary skills to cope with the academic tasks assigned by teachers. Furthermore, the emotional difficulties experienced by the parents of children with NDD have been exacerbated by the COVID-19 pandemic. This means the continuity of family support must be provided to these parents in order to prevent a deterioration of their mental and emotional health. Despite the aforementioned considerations, it is important to note that interventions to enhance homework performance in children with NDD cannot solely focus on emotional or behavioral aspects; instead, they have to incorporate academic measures to address other affected academic domains, such as attention, self-regulation, and academic performance.

At the policy level, the most notable implication is the importance of promoting inclusive policies that are in line with the care and enhancement of mental health in all spheres in which children with neurodevelopmental disorders develop. This may also benefit the family during academic tasks or other challenges in the child’s day-to-day life. It would be beneficial to design early detection programs and provide access to mental health services for students and families that combine educational work, social work, family work, and mental health work.

For mental health professionals, these implications suggest the need to improve communication and collaboration with schools in order to provide comprehensive support to students with neurodevelopmental disorders and their families. Consequently, specific intervention programs need to be developed for both families and students. In addition, education professionals need high-quality training in order to facilitate early detection and intervention.

The present study is subject to a number of limitations. The sample of articles was small, as the publication of studies on the topic is very limited so far. The selected sample combines studies that also included interventions and that did not solely focus on studying the relationship between NDD and homework, which may lead to some variability in the design, methodology, and measures used. Another limitation relates to terminology, as there are differences between countries when referring to special educational needs. Similarly, the term “family involvement” has several facets and considerations [32,33] such as “bonding”, “parent–child interaction”, “parenting practices”, and “academic support”, which should be considered in future research. Finally, although this study was undertaken considering the Overlapping Spheres of Influence model, it would be interesting to address this same research objective using another model or perspective, such as Bronfenbrenner’s ecological model, and therefore consider the infant mesosystem (that is, the interactions between two or more microsystems: family and school, neighborhood, and family, among others). Consequently, this review emphasizes the need for further research to clarify the relationship between neurodevelopmental disorders and homework, with particular consideration of family involvement and the emotional status of both parents and children.

## Figures and Tables

**Table 1:** Bibliometric properties and methodological characteristics of the articles included in the systematic review (N = 11).

Descriptive Variable	Fr	%
Year of publication		
2012	3	27.27
2015	1	9.09
2016	1	9.09
2017	1	9.09
2020	1	9.09
2021	2	18.18
2022	2	18.18
Language		
English	11	100.0
Journal		
Journal of Affective Disorders	1	9.09
Psychology in the Schools	1	9.09
Journal of Clinical Child and Adolescent	1	9.09
Learning Disabilities Research and Practice	2	18.18
Reading and Writing	1	9.09
Journal of Attention Disorders	2	18.18
The Journal of Experimental Education	1	9.09
Journal of Behavioral Education	1	9.09
Remedial and Special Education	1	9.09
Age of the sample		
Elementary Education (6–12 years old)	9	81.82
Secondary Education (12–16 years old)	2	18.18
Sample size		
1–30	2	18.18
31–100	4	36.36
>100	3	27.27
>1000	2	18.18
Sample country		
China	1	9.09
Cyprus	1	9.09
USA	6	54.55
Germany	1	9.09
Israel	1	9.09
Italy	1	9.09
Special educational needs of the sample		
SLD or ADHD	6	54.55
ADHD + comorbidity	2	18.18
Sample not defined	3	27.27
Informant		
Self-report and family report	2	18.18
Self-report, family report, and teacher’s report	1	9.09
Family report	2	18.18
Expert report and family report	1	9.09
Expert report, family report, teacher’s report, and self-report	3	27.27
Intervention tool	2	18.18
Data collection method		
Questionnaires or scales	5	45.45
Questionnaires and psycho-pedagogical test	3	27.27
Questionnaires, psycho-pedagogical tests, and school register	1	9.09
Intervention tool	2	18.18
Total	11	100.0

**Table 2:** Scientific articles on NDD and homework (2012–2022).

Authors (Year)	Sample	Instruments	Conclusions
Booster et al. (2012) [48]	A total of 416 students with monodiagnosis ADHD or comorbid ADHD + internalizing and/or externalizing disorders 5–16 yo	DICA-R ADHD Rating Scale—IV Behavior Assessment System for Children WIAT	- The parents of children with ADHD and a comorbid internalizing/externalizing disorder reported more problems with homework than the parents of children with monodiagnostic ADHD. - The parents of older children (12–16 yo) reported significantly more homework problems than the parents of younger children. - It is important to pay attention to possible comorbidities of SLD for quality interventions.
Katz et al. (2012) [49]	A total of 34 students: 50% with LD Sixth grade + families 11 yo	The Child’s Inventory for Homework Stress The Parent’s Inventory for Homework Stress	- Reducing stress is a necessary but insufficient condition for creating positive emotions and motivation. - The best emotional states for parents and children while doing homework are positive emotions and intrinsic or autonomous motivation.
Mautone et al. (2012) [50]	In total, 91 ADHD students + families + teachers 7–12 yo	HPQ-P HPQ-T HPC PTIQ WIAT-II Homework samples	- Parent-scored student competence was positively related to teacher-rated student competence and academic achievement. - Significant differences exist between children with and without difficulties in the variables studied. - It is important to know the individual characteristics of the students, especially if they present SEN.
Houser et al. (2015) [51]	A total of 24 students with LD 14–16 yo	Radical Raceway (RR) Intervention Program	- By using RR, homework completion and accuracy increased dramatically - In total, 30% more students completed homework daily - When RR was removed, homework completion and accuracy decreased immediately and substantially - RR was an effective way to improve homework completion and homework accuracy
Multhauf et al. (2016) [52]	A total of 39 mothers with their children with LD 7–10 yo	WISC-V The Parenting Stress Index Questionnaire Dyslexia Questionnaire (ad hoc questionnaire)	- Parent training would lead to a reduction in general parental stress, dyslexia-specific stress levels, and an increase in parenting competencies - Mothers who participated in the dyslexia intervention achieved their goals in relation to homework situations
King-Sears et al. (2017) [53]	A total of 19 students: 4 with LD 12–16 yo	Pencasts Intervention Program for homework	- Pencasts improved homework performance - Allows students with LD and non-LD to flexibly use the same technology according to their needs - Pencasts are beneficial for improving learning
Friedman et al. (2020) [54]	A total of 74 students with ADHD-I and LD 7–11 yo	Woodcock–Johnson Test—III CSI COSS The Academic Competence Evaluation Scale The Homework Problem Checklist Child Life and Attention Skills (CLAS) Intervention Program	- The CLAS Program resulted in more benefits in students with ADHD-I monodiagnosis as opposed to students with ADHD-I + LD comorbidity - Supplemental LD-specific intervention is needed in this sample to reduce underlying deficits - Improvements in homework completion were found after the intervention
Touloupis (2021) [55]	In total, 271 families and their children with SLD 10–11 yo	Questionnaire on parental involvement in homework Fear of COVID-19 scale	- The parents of children with LD adopted less beneficial forms of homework involvement - There is high parental fear of COVID-19 and low resilience, which are predictive factors for this type of control-focused involvement - The existence of LD causes parents to be more involved in learning at home
Womack et al. (2021) [56]	A total of 4393 students (474 with IEPs) + families 10 yo	Ad hoc Questionnaire (homework help, IEP, sociodemographic characteristics, satisfaction, communication, and expectations)	- Struggling students experience more difficulty with homework than their peers without educational needs - The parents of children with difficulties are more involved with homework - Parental expectations are lower for students with IEPs - Parents who communicate more with the school are more involved with homework - Parents who are more satisfied with school are less involved with homework
Xiao et al. (2022) [57]	A total of 3993 students: 114 with LD 7–11 yo	Questionnaire on Influencing Factors of Children’s Chinese Reading Ability SCARED DCCC PRS CPSSS CDI-S	- There is a direct association between dyslexia and depressive symptoms, but it is not direct in the case of anxiety symptoms - Stress mediates the relationship between dyslexia and anxiety/depression - Time spent on homework is a mediator between dyslexia and anxiety/depression
Katz et al. (2022) [58]	A total of 108 students (54 with LD) + families 11 yo	BPNSFS The Shame and Guilt Scale HSQ	- Increased levels of stress, frustration, shame, and guilt of parents when involved in the homework of their children with LD - Mediating role of shame and guilt in the parents of children with LD - Shame was related to the stress of parents and children with LD
